# A Large Pure Uterine Lipoma: Its Diagnosis by Pelvic MRI and Histopathology

**DOI:** 10.1155/2019/3929647

**Published:** 2019-12-21

**Authors:** Giannina Calongos, Yoshihiro Ito, Yoko Kubota, Masafumi Handa, Akinori Ida, Yoshiyuki Tsuji

**Affiliations:** Department of Obstetrics and Gynecology, Kobe Adventist Hospital, 8-4-1 Arinodai, Kita-ku, Kobe Hyogo 651-1321, Japan

## Abstract

The patient is a 74-year-old female previously diagnosed with an ovarian tumor at age 55. No changes were noted for one year; however, she was lost to follow-up. Eighteen years later, she presented to a local clinic complaining of diffuse abdominal and flank pain. Abdominal and pelvic ultrasound, CT, and gynecological examination showed a fatty pelvic tumor of approximately 12 cm in diameter. A left ovarian teratoma was suspected, and per the patient's request, she was transferred to Kobe Adventist Hospital for further evaluation and treatment. Pelvic MRI revealed no ovarian enlargement; however, a mass in the uterine body was appreciated with a high signal on T1 and T2 images and signal dropout in the fat suppression images, a finding most consistent with a uterine lipoma. A total hysterectomy and bilateral salpingo-oophorectomy was performed, and histopathological examination confirmed the preliminary diagnosis. No complications were observed during the postoperative period. A pure uterine lipoma is an extremely rare tumor with only a few cases reported worldwide. It is a benign tumor; however, it can sometimes be misdiagnosed as a malignant neoplasm. Pelvic MRI appears to be a useful tool in order to make the correct diagnosis preoperatively.

## 1. Introduction

Lipomatous tumors of the uterus are very rare and have an estimated incidence of approximately 0.03 to 0.2% [1]. Mixed-type fatty tumors containing different amounts of smooth muscle and fibrous tissue have been the most frequent type reported; however, pure lipomas of the uterus are extremely rare [2]. Since only a few cases have been reported, the exact incidence is still not known. Moreover, given the nonspecific findings on pelvic ultrasound and CT [3, 4], they can often be misdiagnosed with other types of neoplasms [5].

The affected patient population consists of mostly postmenopausal women [5]. Uterine lipomas are benign entities and almost always asymptomatic. Malignant conversion of these tumors is rare. Given these characteristics, they are usually diagnosed incidentally [4, 6]; however, they can sometimes present with nonspecific symptoms such as abdominal pain and/or vaginal bleeding [2, 7]. Uterine lipomas are commonly found in the body of the uterus and measure approximately 5-10 cm in size [3, 5, 8]. The differential diagnosis are sarcoma, uterine fatty tumors, and leiomyoma [6].

Here, we report the case of a large pure uterine lipoma preliminary diagnosed via pelvic MRI and confirmed by histopathological examination.

## 2. Case Presentation

A 74-year-old female was transferred to our hospital with a presumptive diagnosis of a left ovarian teratoma. She was diagnosed with an ovarian tumor at age 55 and was followed-up at another hospital with no apparent changes. However, she stopped showing for consult after one year. Eighteen years later, she presented to a local clinic with diffuse abdominal and flank pain. She had a past history of pneumonia and her family history was noncontributory.

**Figure 1:**
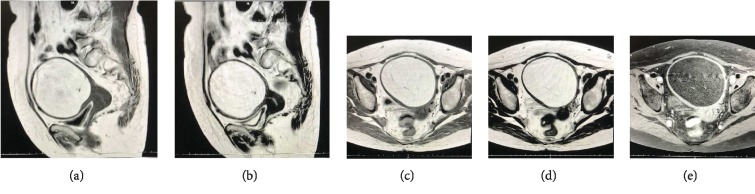
Sagittal (a, b) and axial (c–e) MRI images of the uterine lipoma showing high signal on T1 (a, c) and T2 (b, d) sequences and low signal on fat suppression sequence (e).

An abdominal and pelvic ultrasound and CT showed a fatty pelvic tumor of 12 × 10 cm in size. No lymphadenopathies were observed. Gynecological examination and vaginal ultrasound confirmed the previous characteristics. Serum tumor markers (CEA, CA19-9, and CA125) were within normal levels. Although there were no calcifications nor hairball-like lesions inside the tumor, a left ovarian teratoma was suspected. Per the patient's and family's wishes, she was transferred to our hospital for further evaluation and treatment. A pelvic MRI showed no ovarian lesions, but the uterus was noted to be enlarged to 13 × 11 cm due to a tumor localized in the uterine body. High signal was observed in T1 and T2 images and signal dropout was noted in fat suppression images (Figure 1). As a result, a uterine lipoma was diagnosed.

**Figure 2:**
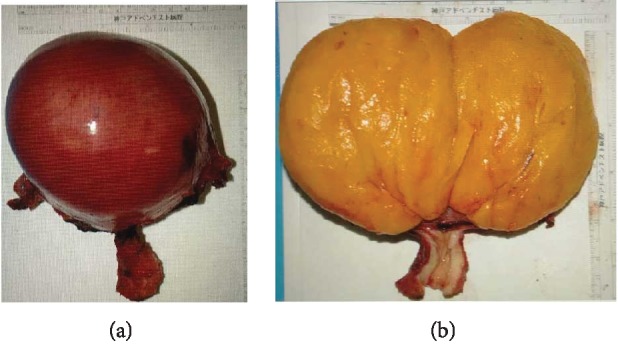
Gross appearance of the uterus showing a well-circumscribed yellow tumor.

**Figure 3:**
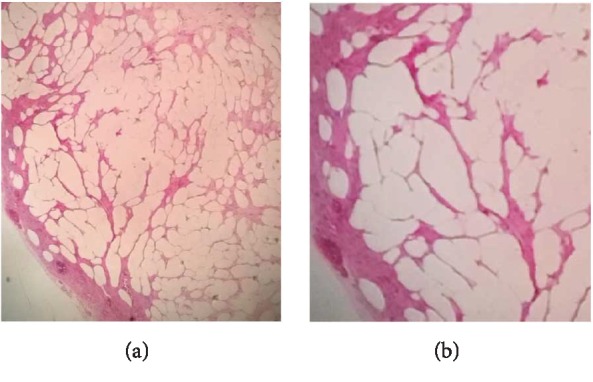
Hematoxylin & eosin staining of the uterine lipoma at ×10 (a) and ×40 (b) magnifications showing mature adipocyte lobules separated by fibrous septae.

After discussion with the patient and her family, an abdominal laparotomy was performed and a 11 × 10 cm tumor originating from the body of the uterus was noted (Figure 2). Both ovaries had normal characteristics. Total abdominal hysterectomy with bilateral salpingo-oophorectomy was performed without complications. Histopathology examination revealed a well-encapsulated tumor composed of mature adipocytes lobules separated by fibrous septae, consistent with a pure uterine lipoma (Figure 3).

The postoperative course was uneventful, and the patient left the hospital 10 days after surgery. No complications were observed after 9 months of follow-up.

## 3. Discussion

Uterine fatty tumors constitute a range of benign tumors that may be composed entirely of adipocytes (pure lipomas) or intermixed with connective tissue or smooth muscle (lipoleiomyomas and lipofibromas) [5]. They are very rare in occurrence and their exact incidence is not yet known. As in our case, the patient population usually affected are postmenopausal women over the age of 50 years [5], with some cases reported in women in their 40s [4]. Uterine lipomas are usually solitary lesions, mostly located in the body of the uterus. They are most intramural as seen in our patient; however, submucosal and subserosal tumors have been described [3, 5].

Although uterine lipomas are difficult to diagnose by imaging, pelvic MRI can be useful in identifying the fatty nature of these lesions [4]. Previous studies have suggested that MRI is the best modality of radiological investigation for preoperative diagnosis [8]. A lipoma of the uterus would appear with high signal on T1 and T2 sequences and show a signal dropout on fat suppression sequences [5]. On ultrasound, these tumors appear as well-defined hyperechoic lesions because of the fat and are avascular with a hypoechoic rim due to the myometrium in the periphery [2]. Due to the tumor location and size, our patient was first suspected to have an ovarian tumor with a thick wall near the uterus. She was subsequently evaluated via ultrasonography and pelvic CT, but the correct diagnosis was made by pelvic MRI.

This type of tumors can present with vaginal bleeding or pelvic discomfort resembling symptoms of leiomyomas [9]. Our patient presented with abdominal pain and a large uterine lipoma was diagnosed. Considering her symptoms and age, an abdominal hysterectomy and bilateral salpingo-oophorectomy was performed. Her recovery after surgery was uneventful, and no abdominal pain was reported during follow-up.

The diagnosis of a pure lipoma of the uterus should only be made by histopathology when smooth muscle cells, if present, are confined to the periphery of the adipocyte lobules [10]. Various theories have been proposed related to the histogenesis of these tumors. Some of them are metaplasia of the smooth muscles or cells of connective tissue, fatty infiltration or degeneration of connective tissue, proliferation of perivascular fat cells, or misplaced embryonic fat cells [11]. However, there is not yet a clear explanation about their origin.

In conclusion, a pure uterine lipoma is a very uncommon benign tumor that can sometimes be misdiagnosed as other neoplasms. We recommend the use of pelvic MRI as a reliable tool in order to make the correct preoperative diagnosis.
